# Anti-nucleosome antibody in sclerodema patients

**Published:** 2011-03

**Authors:** Francisco Gustavo Mendes e Ferreira de Araújo, Thelma Larocca Skare, Renato M. Nisihara, Shirley R. Utiyama

**Affiliations:** * Evangelic University Hospital Medicine Department, Clinical Hospital, Federal University of Paraná, Curitiba, Paraná, Brazil; ** Laboratory of Immunopathology, Clinical Hospital, Federal University of Paraná, Curitiba, Paraná, Brazil

Sir,

Nucleosomes are considered to be the basic element of chromatin. These are formed by 200 ± 40 base pairs segment of DNA wrapped around the (H2A-H2B, H3-H4)_2_ histone octamer with histone H1 bound on the outside^1^. These may become immunogenic, triggering the production of autoantibodies under particular conditions such as presence of drug interactions or infections^1^.

Even though nucleosomes are considered to be the main antigens in the pathophysiology of systemic lupus erythematosus (SLE), some investigators have also found these in systemic sclerosis (SSc). This finding has been an interesting point of discussion because of the discrepancies in results. Wallace *et al*^1^ observed high frequencies of these autoantibodies in scleroderma patients with limited and diffuse form of this disease. Amoura *et al*^2^ detected these in 45.9 per cent of 37 scleroderma patients and Quattrocchi *et al* 3 in 36.3 per cent of 11 patients. On the other hand, Hmida *et al* 4 studying 49 SSc patients found only one positive patient similar to Cervera *et al* 5 who showed one positive in 10 SSc patients. These discrepancies have been attributed to different detection methods used. Binding of anti-Scl-70 to chromatin^6^ and the patient’s ethnic background may also account for these differences.

We studied anti-nucleosome antibodies in 54 consecutive SSc patients in a cross-sectional study at Evangelic University Hospital, Parana, Brazil during February to December 2009 was approved by the local Ethical Research Committee. All 54 investigated patients fulfilled the American College of Rheumatology (ACR) preliminary criteria for SSc^7^. In this group, 40 (7.4%) had limited form, 10 (18.6%) had generalized and 4 (74%) had PM-SSc (scleroderma-polimyositis) form. Patients with lupus mixed features (n=2) were excluded. Four patients were males and 50 female with mean age of 49.2 ± 12.7 yr. Interstitial lung disease was documented in 17 of 52 (32.7%). Anti-Scl-70 antibodies were found in 7 patients (13.4%).

After written consent, blood (5 ml) was collected from each patient. Samples were centrifuged for 10 min at 10000 *g* and serum separated, aliquoted and stored at -80°C until used. The detection of anti-nucleosome antibodies was done by ELISA using nucleosomes extracted from calf thymus chromatin as antigen (Inova Diagnostics Inc, USA). The cut-off point was of 20.0 U/ml, in accordance with manufacturer instructions. Data were analyzed through frequency tables and contingency tables using χ^2^ and Fisher tests with help of the software Graph Pad Prism, version 4.0 and adopting significance of 5 per cent.

Of the 54 patients tested, five (9.2%) were anti-nucleosome positive. Of these five, one was with PM-SSc form and four with limited form and none in the diffuse form. Values ranged between 36 to 151 UI/ml (mean 67 ± 38.8). No association was found between the presence anti-nucleosome antibodies and lung fibrosis or with presence of Scl-70.

In conclusion, our findings show that scleroderma should be considered a possibility while searching for the exact diagnosis of a connective tissue disease in a patient positive for anti-nucleosome antibodies. More research will be necessary to clarify the role of finding these autoantibodies in SSc.
